# Development of Marker-Free Night-Vision Displacement Sensor System by Using Image Convex Hull Optimization

**DOI:** 10.3390/s18124151

**Published:** 2018-11-27

**Authors:** Insub Choi, JunHee Kim, Jisang Jang

**Affiliations:** Department of Architectural Engineering, Yonsei University, 50 Yonseiro, Seodaemun-gu, Seoul 120-749, Korea; insub@yonsei.ac.kr (I.C.); gjjs6645@naver.com (J.J.)

**Keywords:** vision-based displacement sensor, night vision, image convex hull, non-target, dynamic characteristics

## Abstract

Vision-based displacement sensors (VDSs) have the potential to be widely used in the structural health monitoring field, because the VDSs are generally easier to install and have higher applicability to the existing structures compared to the other conventional displacement sensors. However, the VDS also has disadvantages, in that ancillary markers are needed for extracting displacement data and data reliability is significantly lowered at night. In this study, a night vision displacement sensor (NVDS) was proposed to overcome the aforementioned two limitations. First, a non-contact NVDS system is developed with the installation of the infrared (IR) pass filter. Since it utilizes the wavelength of the infrared region and it is not sensitive to the change of a visible ray, it can precisely extract the shape information of the structure even at night. Second, a technique to extract the feature points from the images without any ancillary marker was formulated through an image convex hull optimization. Finally, the experimental tests of a three-story scaled model were performed to investigate the effectiveness of proposed NVDS at night. The results demonstrate that the NVDS has sufficiently high accuracy even at night and it can precisely measure the dynamic characteristics such as mode shapes and natural frequencies of the structure. The proposed system and formulation would extend the applicability of vision sensor not only into night-time measure but also marker-free measure.

## 1. Introduction

In the modern communities, where there are a high population and a high building density in the downtown areas, it is becoming more important than ever to identify and evaluate the status of the existing buildings. As the evaluation of the status of a building is done based on the data containing the building’s behavior information, there is need for a technology that can acquire the behavior data of a building with high reliability. The obtained data of a typical building include the displacement or acceleration. The displacement data are said to be useful for quickly and intuitively evaluating the status of a building through a comparison with the allowable displacement at the design stage. It has been widely utilized in the system identification (SI) technique, which evaluates the status of a building by determining its dynamic characteristics, or in the damage assessment technique, which evaluates the building damage [1,2,3,4,5]. In other words, it is important to acquire the displacement data, which are raw data containing the behavior information of a structure for evaluating its status.

The vision-based displacement sensor (VDS) extracts displacement data by processing the captured image of the structure. It is a non-contact sensor that is easy to install and entails low costs. In general, contact-type sensors such as the linear variable displacement transformer (LVDT) are mostly used to extract displacement data; these contact-type sensors, however, have a limitation: support is needed for the fixed sensor and as such, it is difficult to obtain the displacement data at the point where monitoring is important, such as at the central part of the beam [6]. Other non-contact sensors include the global positioning system (GPS) and the laser displacement sensor (LDS) but they have difficulty extracting the dynamic characteristics, which are closely related to the damage of the structure, as the resolution of GPS is ±10 mm horizontally and ±20 mm vertically [7]. The accuracy of the LDS is about 0.03 mm, which can be applied to the structure but the effective distance for data acquisition is short and support is needed for the sensor [8]. As the VDS, however, does not require a data logger or the support of the part to be measured and can acquire the displacement data of the building from a distance, it can be widely utilized in the existing buildings. The data logger refers to an electronic device that records electronic signals over time of sensors such as LVDS and LDS.

Since the early 1990s, the displacement data required for the health monitoring of structures have been obtained through the VDS. The VDS method was mainly applied to measure the displacement of bridge. Technologies such as algorithms for extracting the feature points from the images in the VDS have been developed in the field of electrical engineering and electronics (Circle Hough Transform [9], Canny Edge Detector [10], Harris Corner Detector [11]). To easily extract the feature points from the images, various markers (circle marker plate, pattern plate, light-emitting diodes [LEDs]) are attached to the area where the displacement of the structure is to be measured [12,13,14,15]. Then the feature points extracted from each image are tracked and the displacement data are obtained by converting the image coordinate system to the physical coordinate system from the geometry of the marker. The analysis that was done in the existing studies verified the reliability of the displacement data obtained from the VDS using the marker and also found that the VDS has a high degree of precision [16,17,18,19,20]. The method of using an artificial marker, however, has disadvantages: it can obtain accurate data only by fixing the marker rigidly onto the structure, there is a possibility that the marker will fall off and only the displacement data of the area to which the marker is attached can be obtained.

In this regard, many studies have been conducted of late to acquire displacement data from images without using a marker. Feng et al. [21,22] conducted a study on the method of extracting displacement data using natural markers such as bolt holes and rivets in structures. As this method, however, can acquire the displacement data only of the area where a natural marker is present in an actual structure, the displacement data of a desired part of the structure cannot be obtained through the VDS. Generally, the method that is used to obtain the displacement data of a structure without using a marker is the digital image correlation (DIC) method, which matches the gray-scale distribution of the image before and after the deformation of the structure to capture the feature points [23,24,25]. According to the research results, the DIC technique has sufficiently higher accuracy than the marker and LVDT, which is a conventional contact-type sensor. Although DIC can handle some of the changes in light intensity, there are needed more applications to solve various time-critical problems [26]. Bartilson et al. [27] proposed a method of extracting displacement data without an ancillary marker, based on MQD (minimum of the quadratic difference) algorithm. While the principle of the MQD algorithm is similar to the DIC technique, the MQD algorithm is an improved method in terms of computational cost because it performs cross-correlation algorithm using fast furrier transform (FFT). The optical flow-based Harris corner detector [11] is a method to extract the displacement from the corner of the structure. According to the related studies [28,29,30]. The structural displacement can be reliably extract without ancillary markers at the desired position of the structure. In recent years, quick and fine movement of the structures can be captured by measuring the displacement using high-speed camera and optical flow-based method [31,32,33]. However, since the basic principle of this method is to maintain the brightness constancy, the accuracy is lowered when the brightness of the light is varied.

The VDS is easier to install than the other displacement sensors and can easily obtain data but the reliability of the data largely depends on natural factors such as the heat waves or camera shake. Especially in the case of the structure, 24-h monitoring is essential but because the input data of the VDS is the wavelength of the visible light region, the VDS has a disadvantage: the reliability of the data is drastically lowered at night, when the visible ray is lacking. Research using LED has been conducted to overcome this disadvantage [34] but it poses problems such as reduction of the accuracy of the displacement data due to the LED light leaks, in addition to the aforementioned problem caused by the marker.

Night vision has been known since 1974, when the U.S. Army began to use an image intensifier to which an image intensification technique was applied. The image intensifier detects part of the infrared region but it is a technology for securing visibility at night only by electrically amplifying the photons in the visible ray region near the green region, which is basically sensitive to the human eye. The infrared camera uses a technology that detects the wavelength of the infrared region through the radiant heat emitted from an object and that converts it to an image. Likewise, although the image intensifier and the infrared camera using the wavelength of the infrared region at night have different basic principles, the two technologies are used to increase the visibility at night. As the equipment used for the image intensifier is expensive, night vision is commonly applied using the infrared camera [35,36]. The image sensors used in general cameras such as the charge-coupled device (CCD) or complementary metal-oxide-semiconductor (CMOS) can detect wavelengths ranging from 400 to 1000 nm, which are the wavelengths of light in the infrared region, including the ultraviolet and visible rays. In other words, as it is possible to detect the infrared region using a general commercial camera, the hardware performance for developing a displacement sensor suitable for night vision with the use of the commercial camera is said to be already available.

The purpose of this study is to propose a night vision displacement sensor (NVDS), which is marker-less VDS optimized for nighttime. The proposed method consists of that for the hardware part, which includes the method of installing the infrared (IR) pass filter of a commercial camera and that for the software part, consisting of a convex hull optimization model that extracts the feature points from the obtained image data without ancillary markers and a scaling factor map for converting the pixel coordinates of the extracted feature points to physical coordinates. The dynamic experiment of a three-story scaled model was performed to determine whether the proposed method extracts highly reliable data in an actual nighttime environment. The dynamic displacement of the model was measured by the NVDS, general VDS with marker and the LDS (the reference data). The displacement data obtained through each measurement method were compared and the dynamic characteristics of the structure obtained from the displacement data were extracted to determine if the proposed method was suitable for monitoring the structure.

## 2. Night Vision Displacement Sensor (NVDS) 

**Figure 1:**
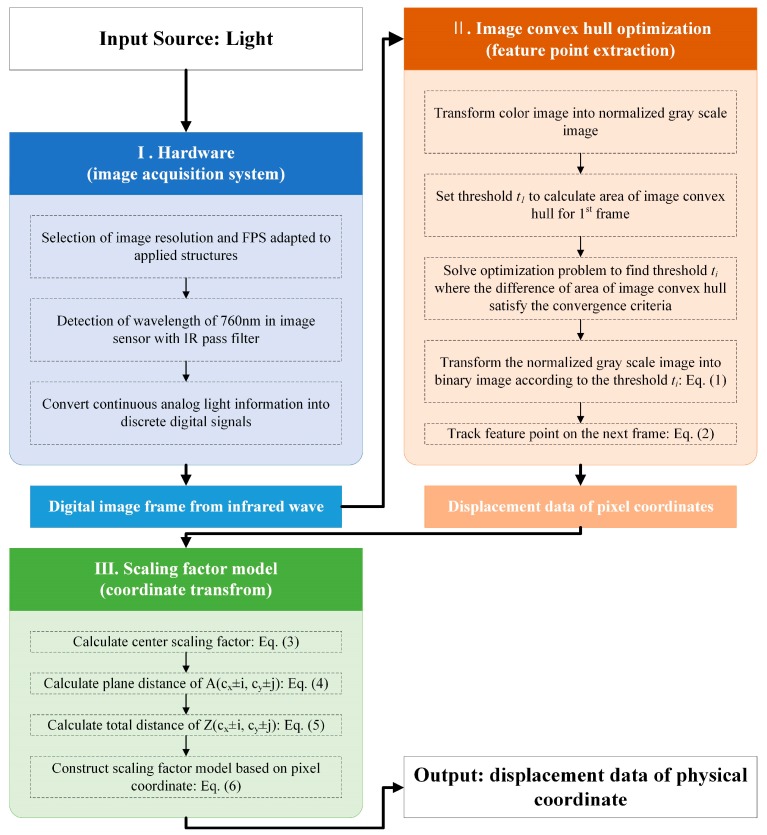
Time domain displacement data acquisition algorithm using night vision optimized displacement sensor (NVDS).

The night vision displacement sensor (NVDS) proposed in this study adopts the technology for extracting the displacement data of a structure using the electromagnetic waves of the infrared region by applying an IR pass filter to a commercial camera. Figure 1 shows the technique for extracting the displacement data using the NVDS. The input data of the NVDS is the infrared ray and the output data is the time domain displacement data of the structure. 

This section consists of three subsections. Each subsection describes the hardware part constituting the NVDS, the feature point extraction part using image convex hull optimization and the scaling factor map converting the coordinates of the extracted feature points.

### 2.1. Hardware Description for Night Vision System

The night vision system consists of a night vision camera, a tripod, a laptop for image processing and a USB cable needed for data communication. The purpose of the image-based measurement system using night vision is to measure the displacement of a structure using the VDS even when the light intensity is low as it is nighttime or due to weather changes. In general, as CCD and CMOS, which are image sensors used in cameras, can detect the wavelength range (more than 700 nm) in the infrared region, it is possible to detect the infrared region using a general camera but a red-eye effect occurs when the infrared region is detected using the general camera. To prevent this phenomenon, an infrared cut-off filter, sometimes called an “IR filter,” is installed between the camera lens and the image sensor to block the infrared region. In contrast, an IR pass filter, which is generally called an “infrared filter,” reflects light with less than 760 nm wavelengths and transmits light with more than 760 nm wavelengths. In this study, the hardware of the night vision measurement system, which detects the infrared region, was configured by removing the existing infrared cut-off filter of a general camera and installing an infrared filter that transmits wavelengths more than those in the infrared region.

**Figure 2:**
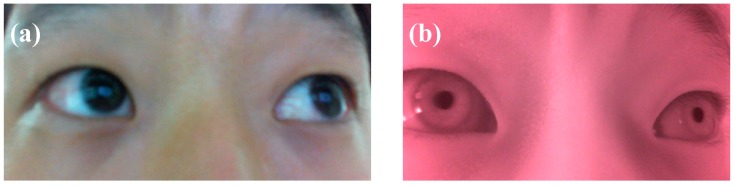
Comparison between on images of a person’s eye taken by commercial cameras; (**a**) LifeCam HD-5000 (general camera), (**b**) LifeCam HD-5000 with IR pass filter (night vision camera).

The night vision camera that was used in this study was LifeCam HD-5000. Figure 2 shows a comparison of the eye region images taken with a general camera and those taken with a night vision camera. All procedures for using Figure 2 were explained to the participant and consent was received to use in advance. As shown in Figure 2, the structure of the iris in the eye region, which was taken with the night vision camera, can be clearly seen but the structure of the iris cannot be clearly seen when taken with the general camera. In addition, as the image taken with the night vision camera received the infrared region, a red-eye effect occurred. As this red-eye phenomenon, however, does not cause image distortion when the image is converted to a gray-scale image through image processing, it was verified that the image of the infrared region can be detected through the night vision system.

### 2.2. Image Convex Hull Optimization Method

A gray-scale image is a set of multiple pixels and each pixel has an integer value ranging from 0 to 255, which corresponds to the intensity of the light. The information of a pixel is greatly influenced by the changes in the external light but it changes according to the shape and structure of an object. The method of recognizing a human face or an object using the information of the pixel has been widely used in the field of computer vision. For extracting the displacement data of a structure from the structure image taken without using a marker, however, or for extracting the displacement data of the desired position from the image, the method of extracting the displacement data through the shape information of a structure without using accessories such as a marker is said to be the most ideal. This study developed an image convex hull optimization method that can extract the same image convex hull from every frame of the image taken from the structure, so that the displacement of the structure could be extracted without using any marker.

#### 2.2.1. Image Convex Hull

**Figure 3:**
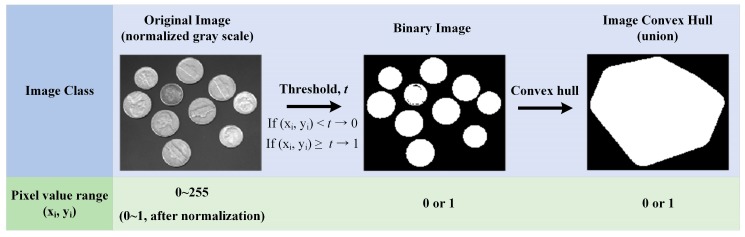
Description of image convex hull.

The image convex hull is a convex polygon corresponding to an area filled with white dots (the parts represented by 1 in a binary image) due to the image binarization of a gray-scale image into specific threshold values ranging from 0 to 1, as shown in Figure 3. In the field of computer vision, various image processing algorithms are used in the image classification method to classify objects from their recognized feature points and to identify them [37,38]. Among these, the image convex hull was used in this study because it facilitates the extraction of specific shapes or feature points from the image and has high computational speed.

To facilitate the definition of the image convex hull, it is necessary to normalize the gray-scale image with values ranging from 0 to 255 and to set the pixel value to 0–1. The reason for the need to go through the normalizing process is that the range of the threshold values that distinguish the boundaries of the binary image can be reduced to the range of 0 to 1, not 0 to 255. Then the threshold value is calculated by analyzing the histogram on the brightness of the image and the image is binarized by setting the value of the pixel with a value higher than the threshold to 1 and that with a value lower than the threshold to 0.

In the binary image, the convex hull filled with white dots can be defined and as such, the image convex hull can be obtained based on this. That is, the image convex hull is obtained from the binarized image obtained from the threshold value and therefore, the shape of the image convex hull can be determined by adjusting the threshold value [39].

#### 2.2.2. Formulation of Convex Hull Optimization for Extracting Feature Points

As various binary images can be obtained by adjusting the threshold value, a variety of image convex hulls can be obtained from one image. In this study, we use the centroid of image convex hull as the feature point and extract the displacement data through the centroid change to extract the feature point from the image of a structure taken without using a marker. If the same convex hull can be extracted from each frame by adjusting the threshold value, the centroid of the convex hull that determines the displacement data of the structure can be precisely extracted. The image convex hull optimization method was used in this study to find the image convex hull that is the same as the image convex hull of the reference frame by adjusting the threshold value in every frame. In the study, the reference frame was selected as the first frame of the image.

To find the same image convex hull as the reference frame in every frame, a measure is needed to compare the image convex hull extracted from each frame and the image convex hull of the reference frame. Even though there may be various comparative measures, a quick comparison of similarities can be done by determining the similarity of the image convex hull of the two frames through the area of a figure. If the brightness of the light changes drastically, or if severe deformation of a structure occurs, it is difficult to find the same image convex hull. Images, however, are usually taken at 30 frames per second and the night vision used in this study is not sensitive to the visible light region and thus has no trouble finding the same convex hull. The validation process of the proposed method of finding feature points and its results will be discussed in detail in Section 2.2.3. If the difference between the image convex hull area of the reference frame and the area of the image convex hull in the *i*-th frame converges to a certain convergence condition, it is thought that the same image convex hull was found. Therefore, the image convex hull optimization problem can be defined as follows:

$$\begin{array}{c} \mathrm{Find}\;t_{i}\\ \mathrm{Min}\;\left|A{\left(t_{1}\right)}_{1}-A{\left(t_{i}\right)}_{i}\right|\\ s.t\;0\leq t_{i}\leq 1,\;i=2,\;3,\;\cdots ,\;n \end{array}$$

where, $t_{i}$ is the threshold value in i-th frame, $A_{i}$ is the area of image convex hull in i-th frame and n is number of image frame.

The process of extracting the feature points from the formulated convex hull optimization problem is shown below.

1. The region of interest (ROI) of the reference frame is set to determine *t*_1_, which is a threshold value for defining the image convex hull for this ROI. The threshold value *t*_1_ of the first frame is obtained through Otsu’s method [39], which can minimize the difference in the numbers of black and white pixels by analyzing the histogram.
2. The convergence criterion needed for the image convex hull optimization problem is set. The convergence criterion can be determined by considering the distance between the camera and the target, the pixel pitch of the camera and the ROI. In this study, when the convergence criterion was set to more than 10^−9^ m^2^, which is the area projected in the image sensor, the errors in the extraction of the feature points were reduced.
3. The optimization problem is solved by adjusting the *t_i_* value of the *i*-th frame. If the area of the convex hull meets the convergence condition when compared with the value of the reference frame, the *t_i_* value is stored and then the process is repeated for the next i + 1-th frame.
4. Once the *t_i_* value is obtained for all the frames, the image is binarized based on the *t_i_* value and a convex hull filled with white dots is obtained in the binarized image. The centroid is calculated from the obtained convex hull to extract the feature points and the feature point coordinates (*u_i_*, *v_i_*) of the *i*-th frame are stored. The above process is repeated for all the frames.
5. With respect to the coordinates of the obtained feature points, the relative coordinates (*U_i_*, *V_i_*) are obtained using Equation (1). The obtained relative coordinates are converted to physical coordinates in pixel units using the scaling factor model that converts the coordinates discussed in Section 2.3.

(1) $$\left(U_{i},\;V_{i}\right)=\left(u_{i+1},\;v_{i+1}\right)-\left(u_{i},\;v_{i}\right)$$

where the range of the *i* value is from 1 to n − 1 and *n* is the total number of frames.

Through the above process, the feature points of a structure can be extracted without using a marker and the displacement data can also be extracted therefrom.

#### 2.2.3. Validation of Convex Hull Optimization

As the image convex hull optimization algorithm is a method of extracting the same shape information in every frame using the area, which is the structural shape information, there is a need to verify the convergence characteristics, including the setting of the appropriate convergence criterion for the area. The convergence criterion can be calculated differently depending on the size of the camera’s image sensor, the distance from the target and the focal length. Considering the focal length, distance and pixel pitch of the camera that was used in this study, the convergence condition was determined to be less than 10 × 10 pixels in the difference of the areas between the first frame (the reference frame) and the corresponding frame. If this convergence condition is converted to the units of the actual physical coordinates, it will show an area difference of 1.11 × 10^−9^ m^2^. Therefore, the same shape information with almost no difference from the area of the reference frame can be extracted.

**Figure 4:**
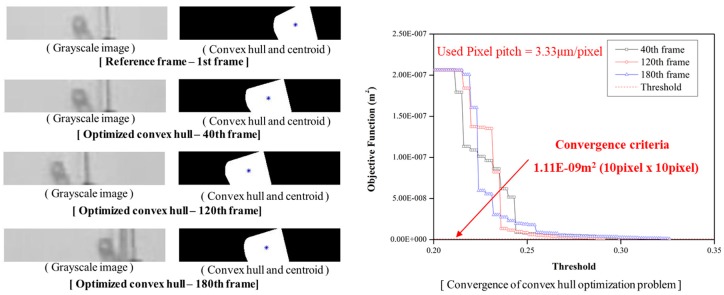

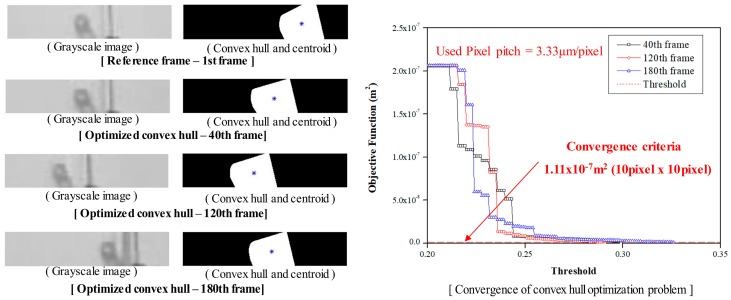
Gray scale image and its convex hull to extract feature point; Convergence of image convex hull optimization method for specific convergence criteria

Figure 4 shows the shape information and feature points obtained through the image convex hull optimization; the gray-scale image in the 40th, 120th and 180th frames, which are arbitrary frames; and the shape information of the first frame, which is the reference frame. In addition, a graph showing the convergence of the objective function of the optimization algorithm according to the threshold value reveals that the objective function converges so that the area difference can be minimized in all the frames. Based on this results, the convergence of the image convex hull optimization was confirmed and it was proven that the shape information of the actual structure could be extracted precisely.

The proposed image convex hull optimization algorithm and Lucas-Kanade Optical flow algorithm [40] are similar in that there is no need for an ancillary marker to obtain the displacement data from the image. Lucas-Kanade optical flow algorithm is a way to solve the basic optical flow equation for all the pixels in that neighborhood by the least squares criterion. The definition of optical flow is apparent motion of brightness patterns, so the optical flow can be said the projection of the three-dimensional velocity vector on the image. Therefore, the brightness constancy must be maintained to extract the displacement data of structure using the Lucas-Kanade algorithm. If the brightness constancy is not maintained, the reliability of the displacement data obtained from the Lucas-Kanade algorithm is drastically decreased [41]. On the other hand, the proposed image convex hull optimization technique has an advantage in that it is not sensitive to the change of light brightness unlike the Lucas-Kanade algorithm, because the feature point is extracted by comparing the shape information obtained by changing the threshold value of the image sequence with the predetermined shape information of the reference frame.

### 2.3. Scaling Factor Model

The scaling factor converts the pixel coordinates of the feature points to physical coordinates. The scaling factor (*SF*_1_) obtained using a marker can be calculated based on the ratio of the actual marker size to the size of the marker in the image, as follows:

(2) $$SF_{1}=\frac{D_{1}}{d_{1}}$$

where, $D_{1}$ is the diameter of a maker in physical plane (mm) and $d_{1}$ is the diameter of a maker in the image plane (pixel).

When a marker is not used, the scaling factor (*SF*_2_) can be calculated from the distance Z from the camera and the target, the focal length f and the pixel pitch R of the image sensor, as shown below.

(3) $$SF_{2}\left(cx,cy\right)=\frac{Z}{f}R$$

Ideally, the *SF*_1_ calculated from actual size of the marker and the *SF*_2_ calculated from the external condition should yield the same result. In preliminary experiment, it was observed that the error between *SF*_1_ and *SF*_2_ increases as the marker was moved away from the center of the image plane. Due to the characteristics of a pinhole camera, while the distance between the marker and the camera obtained through the camera varies depending on the position in the image (or the position of the actual target), the focal length is constant. That is, if the marker or object moves away from the center of the image plane, the distance *Z* from the camera increases, which needs to be corrected. In this regard, the scaling factor map, in which the scaling factor is calculated differently depending on the pixel coordinates in the image plane of the object, can be obtained using the equations below.

(4) $$A\left(cx\pm i,cy\pm j\right)=\sqrt{(i\times SF_{2}{\left(cx\pm i\mp 1,cy\right)}^{2}+(j\times SF_{2}{\left(cx,cy\pm j\mp 1\right)}^{2}}$$

(5) $$Z\left(cx\pm i,\;cy\pm j\right)=\sqrt{Z{\left(cx,cy\right)}^{2}+A{\left(cx\pm i,cy\pm j\right)}^{2}-2Z\left(cx,cy\right)A\left(cx\pm i,cy\pm j\right)\cos\left(\theta \right)}$$

(6) $$SFM\left(cx\pm i,\;cy\pm j\right)=\frac{Z\left(cx\pm i,cy\pm j\right)}{f}R$$

where, A is the distance, *c_x_* and *c_y_* is center pixel of image plane and *SFM* is scaling factor model.

The camera that was used in this study was LifeCam HD-5000, which has 30 fps at the 1280 × 760 resolution and a 24 mm focal length. At the 1280 × 760 resolution, the cx and cy values are 640 and 380, respectively. The R value is a pixel pitch, which is the size of the actual sensor occupied by one pixel in the image plane and the R value is 3.33 μm/pixel when LifeCam HD-5000 is used to make a video. For more information about scaling factor map, see the reference [42]. 

## 3. Shake Table Experimental Outline

The objective of the shake table experiment is to verify the accuracy of the dynamic displacement of a structure obtained using an NVDS at night. Through a preliminary experiment, the static displacement was precisely extracted using the NVDS and it was proven through the shake table experiment that the dynamic displacement and characteristics of a structure could be accurately measured using the NVDS. A scaled model of three-story one-span shear frames was fabricated and used in the shake table experiment. The dimensions of the scaled model were 150 mm in the short-side direction and 250 mm in the long-side direction and the height of each story was 400 mm. The section that was used in the scaled model was steel SS400. The cross-sectional size of the column was 5 × 5 mm, that of the beam was 4 × 6 mm and that of the brace was 4 × 6 mm. The slab was made of 5-mm-thick acrylic and was assembled using bolts. Brackets for installing the brace were welded onto both ends of the column and the brace could be mounted and disassembled using bolts. The weight of each story was 3.03 kg, including the 2 kg mass plate and the weight of the whole model was 9.09 kg.

**Figure 5:**
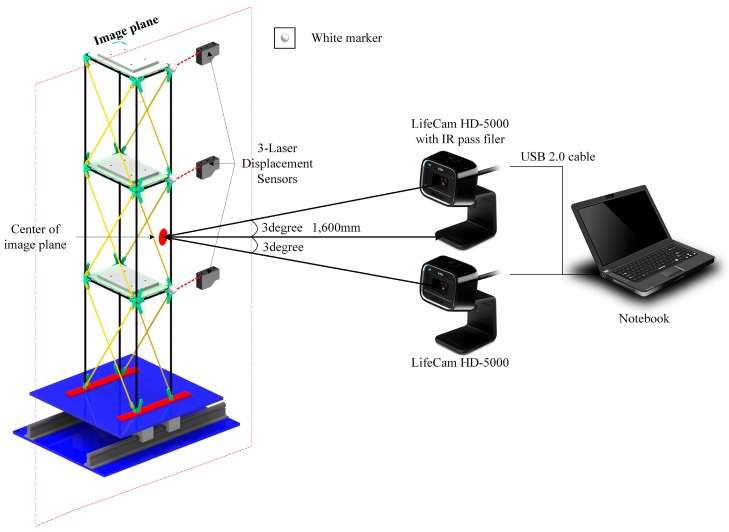
Shake table experimental setup of scaled model with dynamic displacement measuring system.

As shown in Figure 5, a brace was installed in the long-side direction of the scaled model to prevent out-of-plane behavior and a load was applied using a shake table in the short-side direction. For the load, 50 Hz-bandwidth white noise was used and the amplitude of the load that was applied for the structure to behave in the elastic section was scaled down based on the analysis of the scaled model before the experiment. The frequency components of the white noise had 0.0977 Hz intervals from 0 to 50 Hz and this suggests that the frequency band was sufficient for detecting the dynamic characteristics of the scaled model.

There are three methods of measuring the dynamic displacement data of the scaled model: that using an LDS, that using an NVDS and that using a VDS with a marker. The accuracy of the displacement data obtained from the NVDS at nighttime was evaluated by comparison with the displacement data obtained using the other methods. In addition, the displacement data obtained using an LDS was utilized as reference data.

**Figure 6:**
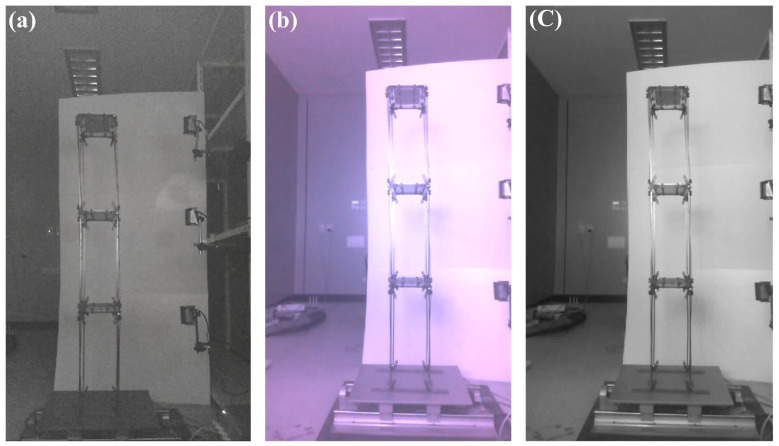
Real images of scaled model using shake table experiment taken into (**a**) general LifeCam HD-5000, (**b**) LifeCam HD-5000 with IR pass filter, (**c**) gray-scale image of LifeCam HD-5000 with IR pass filter.

The experiment was conducted at 10:00 p.m., when there was low light intensity. Figure 6 shows a comparison of the photographs taken with LifeCam HD-5000 equipped with an IR pass filter and those taken with a general camera. As shown in Figure 6, the images taken with the general camera are dark overall due to insufficient light intensity, whereas those taken with the camera using an IR pass filter are clear despite the fact that they were taken at a time when there was low light intensity. That is, in the case of the general camera, noise is generated in the image due to the insufficient light intensity at night. In the case of the camera to which an IR pass filter was applied, however, as the image is generated by receiving the wavelengths in the infrared region even at night, less noise is generated than in the general camera. The image was taken at a 1280 × 760 resolution (100 megapixels) and at 30 fps. The distance between the camera and the center of the structural image was 1600 mm, the focal length was 24 mm, the angle was 3° and the scaling factor calculated through the scaling factor model from the external environment and camera specifications was 0.2219–0.2236 mm/pixel, with a 5-subpixel level.

## 4. Test Results of Proposed NVDS

This chapter analyzes the reliability of the dynamic displacement obtained from the NVDS. To analyze the reliability of the dynamic displacement data, the displacement data was directly compared with that obtained using an LDS (the reference data). In addition, the natural frequencies and mode shapes of the scaled models were extracted using the obtained displacement data and these were compared with the analytical values using the commercial program MIDAS-Gen.

### 4.1. Dynamic Displacement

**Figure 7:**
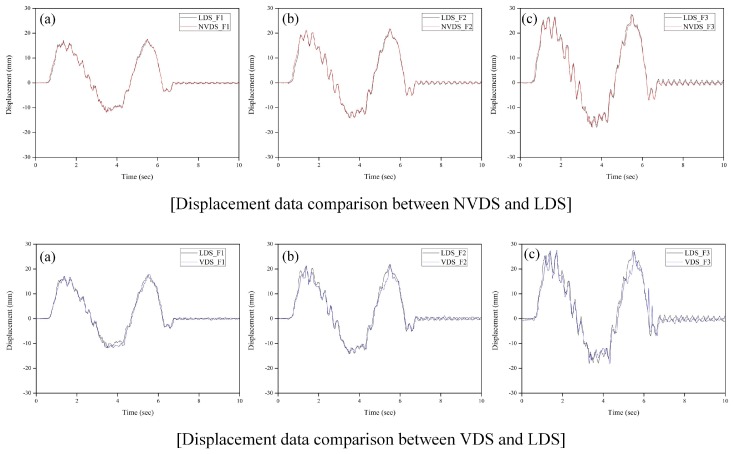
Dynamic displacement comparison between LDS, NVDS and VDS. (**a**) 1st floor, (**b**) 2nd floor, (**c**) 3rd floor.

The dynamic displacement data obtained from the NVDS was directly compared with the reference data obtained using the LDS to analyze the reliability. Figure 7 shows a time-displacement graph that compares the displacement data of the scaled model to which white noise had been applied obtained using the NVDS and that obtained using the LDS. The displacement data of the scaled model obtained using the NVDS shows that the amplitude and period of the data are in good agreement with those of the displacement data obtained using the LDS. In addition, it was confirmed that the NVDS could precisely measure not only the large displacement occurring in a structure under loading but also the relatively small displacement of about 2 mm that occurs during the free vibration after loading. On the other hand, the VDS was similar to the LDS in terms of the maximum and minimum displacements but it failed to extract the displacement data due to the vibration in the structure’s high-frequency region. This means that in the case of the VDS, the reliability of the displacement data was low at night, when there was low light intensity, even when a marker ensuring easy identification was used.

The pixel coordinates were converted to physical coordinates through the scaling factor map calculated from the distance between the camera and the scaled model, the angle and the focal length. The scaling factor value at the center of the image was 1.1095 mm/pixel and that at the end of the image was 1.1180 mm/pixel. In addition, the revolution of the TVDS was 0.2219–0.2236 mm/pixel because 1 pixel was divided into 5 subpixels in the existing image in the image processing. If the subpixel level is increased, the accuracy of the sensor may be improved as the resolution of the NVDS is lowered. As the number of pixels to be computed increases, however and as the pixel information of the existing image may be distorted, a moderately regulated level is required. In this experiment, as shown in Figure 7, the displacement data obtained using the NVDS was found to secure a sufficient sensor resolution even when compared with the displacement data obtained using the LDS with a 0.01 mm resolution. 

For reliability analysis, the displacement data obtained from the NVDS was numerically compared to that obtained from the LDS through root mean square (RMS) analysis and the results are summarized in Table 1. The RMS difference between the LDS and the NVDS was 0.53% on the first story, 0.24% on the second story and 2.51% on the third story. Even though the largest error occurred on the third story, the numerical error between the two data did not exceed 3%. That is, it was confirmed that even if there are no natural or artificial markers in the structure, highly reliable dynamic displacement can be obtained using the proposed NVDS.

### 4.2. Modal Analysis 

**Figure 8:**
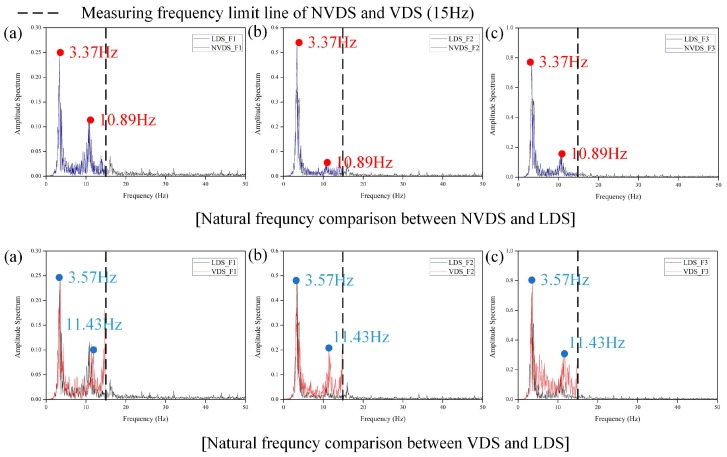
Comparison of natural frequency between LDS and NVDS and between LDS and VDS. (**a**) 1st floor, (**b**) 2nd floor, (**c**) 3rd floor.

If the obtained displacement data can be used to extract the dynamic characteristics of the structure, it can also be used for system identification or damage assessment. Therefore, it can be said that it is also important to analyze the reliability of the obtained dynamic characteristics to verify the performance of the sensor. The dynamic characteristics of the structure, such as the mode shape and the natural frequency, were extracted by converting the time domain displacement data to the frequency domain. Figure 8 shows a graph that compares the natural frequencies obtained from the LDS, NVDS and VDS. The first natural frequencies obtained from the LDS and NVDS were the same (3.37 Hz) and the second natural frequencies were also the same (10.89 Hz). The first natural frequency obtained from the VDS, however, was 3.57 Hz and the second natural frequency was 11.43 Hz, which were different from those obtained from the LDS and NVDS. Even in the results of the comparison of the displacement data, as the reliability of the displacement data obtained using the VDS was low, it was confirmed that for the analysis of a structure’s dynamic characteristics, the reliability of the NVDS, which extracts the displacement data through the infrared region’s light intensity information, at night is higher than that of the VDS. The analysis showed that the NVDS could not measure the natural frequency for the third mode of the structure due to aliasing effect. The aliasing effect means that if the sampling rate is lower than the actual signal rate, which mean the natural frequency of the structure in this case, the measured frequencies differs from the actual natural frequencies. Since the sampling rate of the NVDS is 3.3 ms, it is possible to analyze only the frequency range of up to 15 Hz. The third natural frequency of the structure obtained using the actual LDS was 16.10 Hz, which exceeded the measurement range of the NVDS; thus, the third natural frequency could not be extracted using the NVDS.

To verify the accuracy of the dynamic characteristics obtained from the displacement data, the scaled model was modeled using MIDAS-Gen, a numerical analysis model and then the natural frequencies were compared. The results are summarized in Table 2. The difference between the natural frequencies obtained using the numerical analysis model and those obtained using the LDS and NVDS was less than 2% but the VDS showed a maximum error of 7.58%. In other words, the NVDS is thought to precisely extract the first and second natural frequencies of a structure to a level similar to that with the LDS and it is considered that its performance is equivalent to that of the LDS, which was shown in the experiment herein to have a high degree of precision.

**Figure 9:**
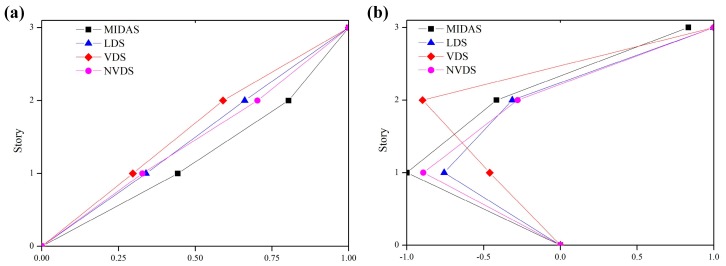
Mode shape comparisons between analytical model and experimental value (**a**) 1st mode shape; (**b**) 2nd mode shape

**Figure 10:**
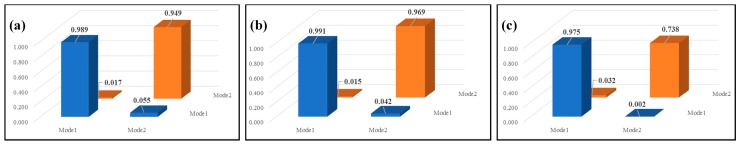
Modal assurance criterion analysis results between analytical model and experimental value; (**a**) MIDAS and LDS; (**b**) MIDAS and NVDS; (**c**) MIDAS and VDS.

Figure 9 is a graph showing a comparison of the mode shapes obtained using the LDS, NVDS and VDS and those analyzed using MIDAS. The first-order and second-order mode shapes obtained using the LDS and NVDS were similar to those of MIDAS but the second-order mode shape obtained using the VDS was very different. In addition, model assurance criterion (MAC) analysis was performed to quantitatively analyze the associations between the mode shapes. The closer the MAC value is to 1, the greater the similarity between the two mode vectors. As showing Figure 10, the MAC analysis results showed that the measuring technique that can obtain the mode shape most similar to that obtained using MIDAS was that using NVDS, which obtained the most accurate mode shape although it had only a slight difference from that obtained using the LDS. In the case of the VDS, as the first-order mode shape was 0.975, the similarity degree was close to 1. The second-order mode shape, however, was 0.738, showing a difference from 1 in the degree of similarity. Therefore, it can be said that the second-order mode shape obtained using the VDS had a low degree of reliability. In other words, the proposed NVDS is capable of precisely extracting the displacement data of a structure even at nighttime and is suitable for use in the health monitoring of a structure.

## 5. Conclusions

In this study, a night vision displacement sensor (NVDS) system was proposed to measure the displacement of structure without any ancillary markers even at night-time. In terms of the hardware, the key technology of the NVDS is the night vision camera to which an infrared (IR) pass filter was applied. In terms of the software, its key technology is the image convex hull optimization algorithm, which extracts the feature points without using natural markers like bolt holes in a structure or artificial markers attached to the structure. The reliability of the displacement data obtained using the NVDS was verified experimentally through an excitation test of a three-story scaled model. The results of the study are summarized as following.

1. Night vision camera applying IR pass filter to a commercial camera can extract information in the infrared and is not greatly affected by visible ray. While the general commercial camera could not capture the structure of the iris in the eye region, the night vision camera could extract the information of the iris. Also, the noise of the image taken with the night vision camera was small during the night but the noise of the image taken with the general camera was large.
2. The image convex hull optimization algorithm to extract feature points without ancillary markers was formulated and verified by convergence analysis. The difference in convex hull area obtained through each image is 1.11 × 10^−9^ m^2^ or less, so this proves that the feature points can be defined as the centroid of the convex hull in every image frame.
3. The results of the excitation test of the scaled model at night-time showed that the displacement data were precisely measured from consecutive image without marker using the NVDS. The difference in the RMS values between the displacement data obtained using the NVDS and that obtained using the LDS (the reference data) was 0.24–2.51%. This is acceptably small; hence it experimentally proves that the dynamic displacement of a structure can be reliably obtained using an NVDS even at night.
4. The dynamic characteristics of the structure were analyzed using the displacement data obtained using the NVDS. The analysis results showed that the NVDS was able to precisely measure the structure’s natural frequency. According to the MAC analysis, the mode shape obtained using the NVDS was very similar to the experimental mode shape obtained using the LDS and the analytical mode shape obtained based on the numerical analysis results. In other words, the proposed NVDS is capable of precisely measuring not only the displacement data but also the dynamic behavior of a structure.

The NVDS developed in this study was able to accurately measure the displacement of a desired part of a structure through the night vision camera and the image convex hull optimization technique without ancillary marker, even at night-time. In addition, the dynamic characteristics of a structure, such as the mode shape and the natural frequency, can be accurately measured using the NVDS. Therefore, the proposed NVDS might be considered as an alternative of existing sensors, especially suitable for the structural health monitoring with less visible light.

## Figures and Tables

**Table 1:** Displacement data reliability results in the white noise test.

Story	RMS (mm)		
	LDS	NVDS	VDS
1	7.0681	7.0303 (0.53%)	7.2684 (2.83%)
2	8.6418	8.6625 (0.24%)	8.0935 (6.34%)
3	10.9697	10.6947 (2.51%)	10.1105 (7.83%)

**Table 2:** Displacement data reliability results in the white noise test.

No. of Mode	Analytical Model (Hz)	Test Result (Hz)		
		LDS	NVDS	VDS
1	3.31	3.37 (1.81%)	3.37 (1.81%)	3.57 (7.58%)
2	11.08	10.89 (1.71%)	10.89 (1.71%)	11.43 (3.16%)
3	17.57	16.10 (8.37%)	-	-
